# Supplementary figures and images for: Noise improves the association between effects of local stimulation and structural degree of brain networks
Source: PLoS Comput Biol. 2023 May 11;19(5):e1010866. doi: 10.1371/journal.pcbi.1010866 (PMC10205011; doi:10.1371/journal.pcbi.1010866)

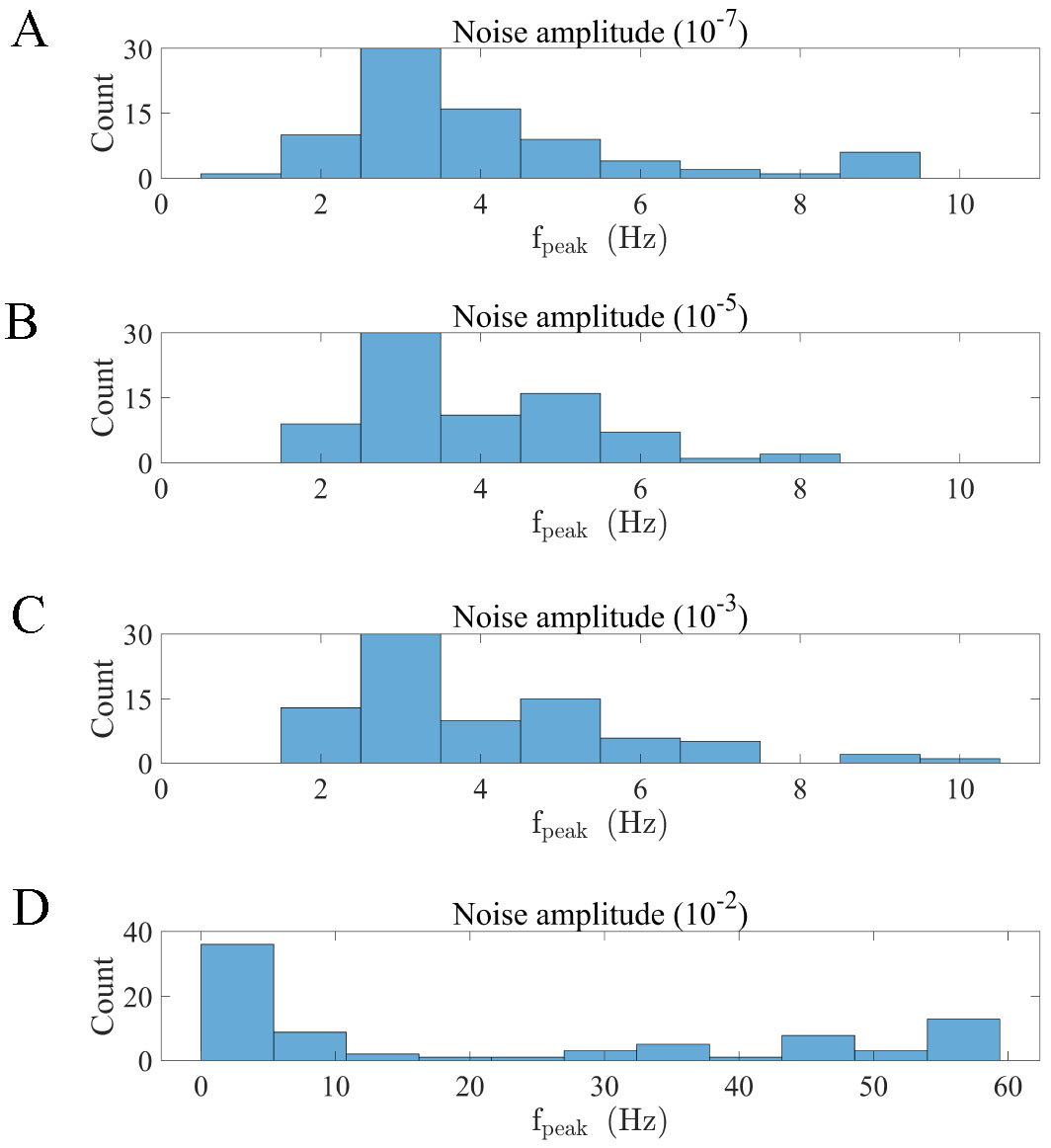

Supplement: S1 Fig — (A) Noise amplitude = 10−7. (B) Noise amplitude = 10−5. (C) Noise amplitude = 10−3. (D) Noise amplitude = 10−2. Panels (A-C) show similar distributions, remarkably different from that of panel (D). These results are comparable to Fig 2C. (JPG) [file pcbi.1010866.s001.jpg]

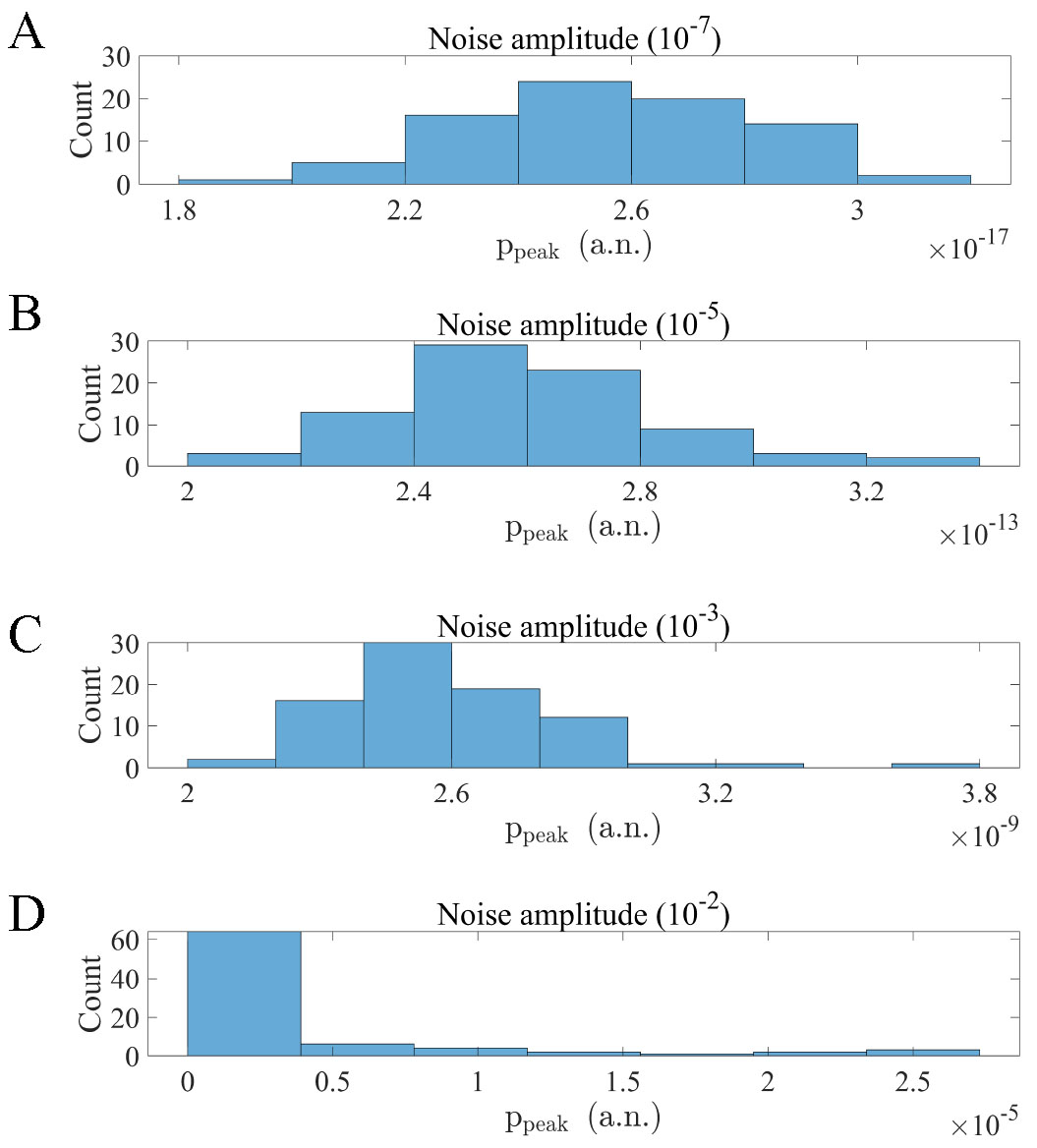

Supplement: S2 Fig — (A) Noise amplitude = 10−7. (B) Noise amplitude = 10−5. (C) Noise amplitude = 10−3. (D) Noise amplitude = 10−2. Noise amplitude increases the peak power in all brain regions, consistent with the results in Fig 2D. (JPG) [file pcbi.1010866.s002.jpg]

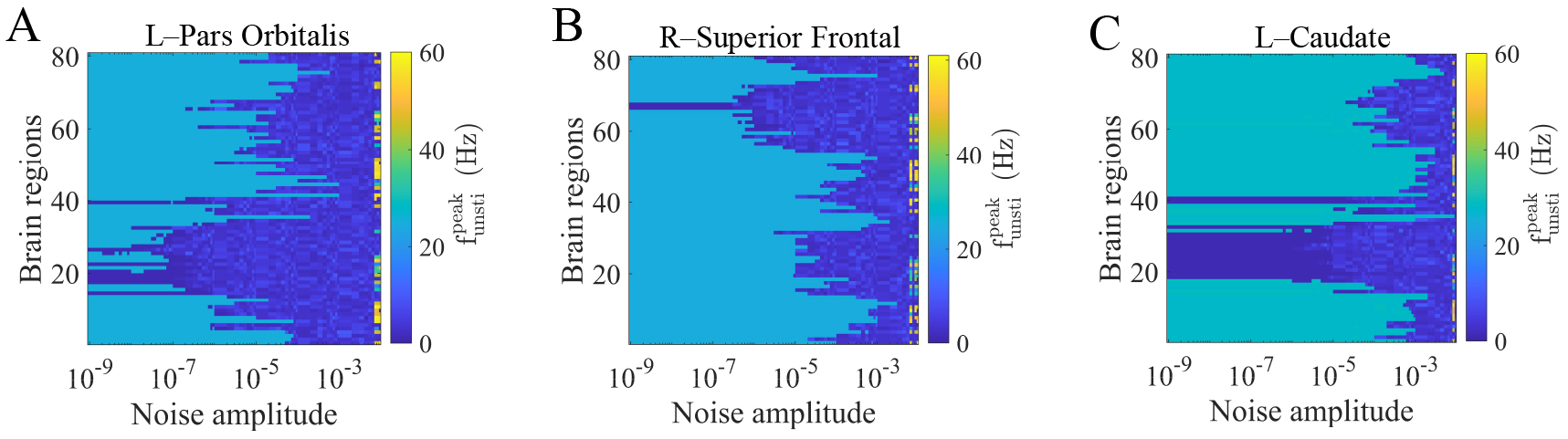

Supplement: S3 Fig — (A) L-Pars Orbitalis (small degree). (B) R-Superior Frontal (moderate degree). (C) L-Caudate (large degree). (JPG) [file pcbi.1010866.s003.jpg]

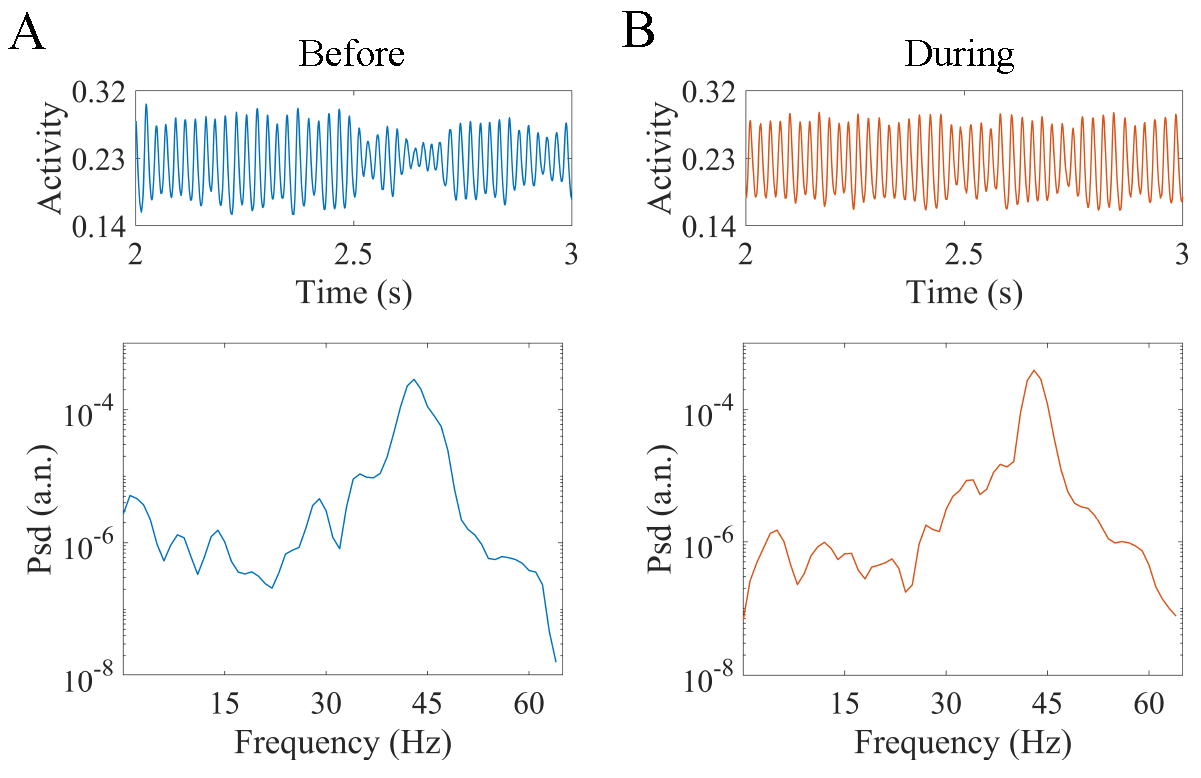

Supplement: S4 Fig — In this realization, the unstimulated region R-Superior Parietal is already in the oscillatory state before stimulation. Results show that the high peak frequency is almost unaffected by stimulation. The upper panels of subfigures show the time series of the R-Superior Parietal region when stimulating the L-Pars Orbitalis region under a large noise amplitude (10−2). The lower panels of subfigures show the power spectra of the corresponding time series. (A) Before stimulation. (B) During stimulation. (JPG) [file pcbi.1010866.s004.jpg]

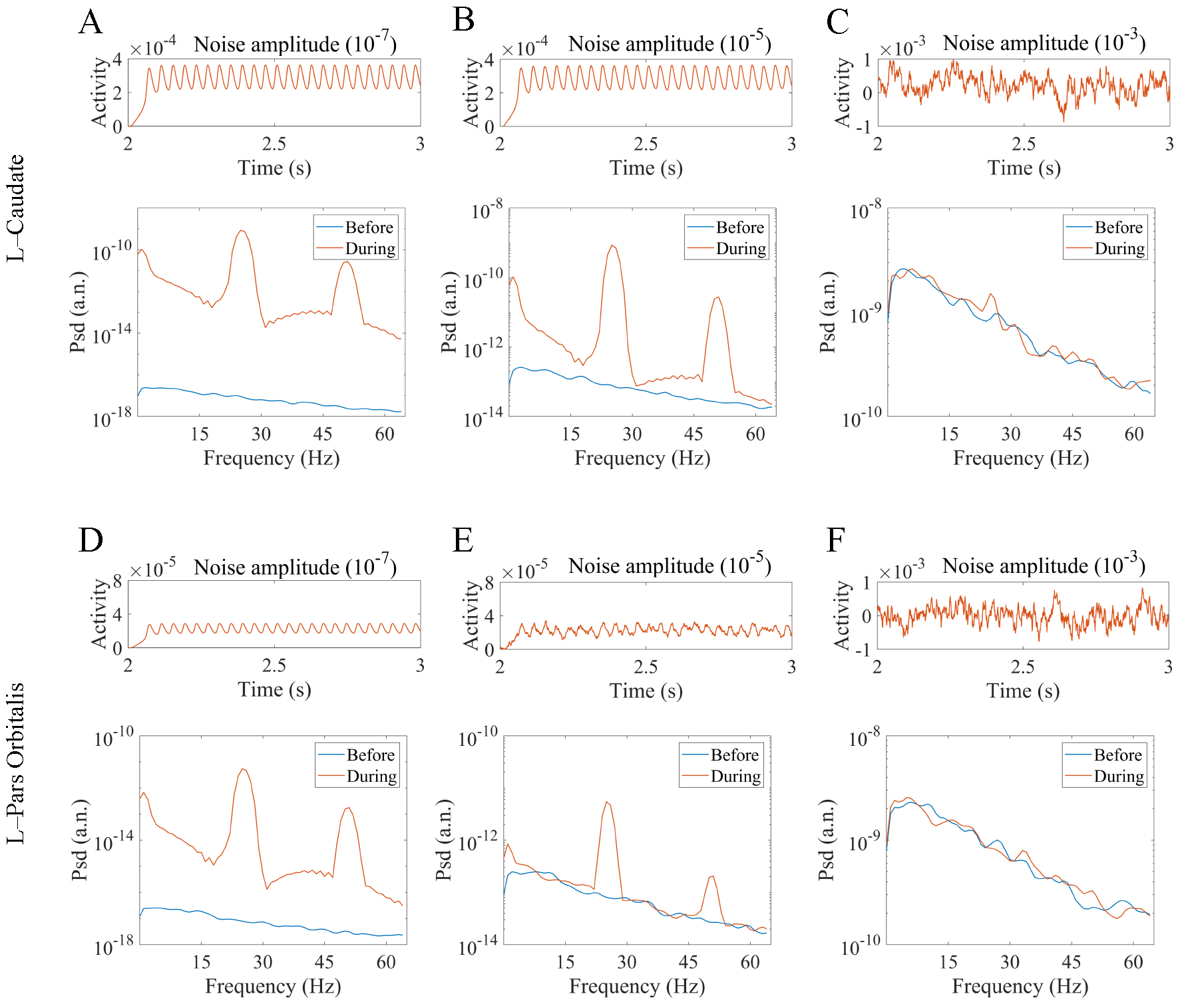

Supplement: S5 Fig — The upper panels of subfigures show the time series of two unstimulated brain regions at different noise amplitudes when stimulating the R-Lateral Orbitofrontal region. The lower panels of subfigures show the power spectra before (blue) and during (orange) stimulation in the corresponding condition. (A) L-Caudate, noise amplitude = 10−7. (B) L-Caudate, noise amplitude = 10−5. (C) L-Caudate, noise amplitude = 10−3. (D) L-Pars Orbitalis, noise amplitude = 10−7. (E) L-Pars Orbitalis, noise amplitude = 10−5. (F) L-Pars Orbitalis, noise amplitude = 10−3. (JPG) [file pcbi.1010866.s005.jpg]

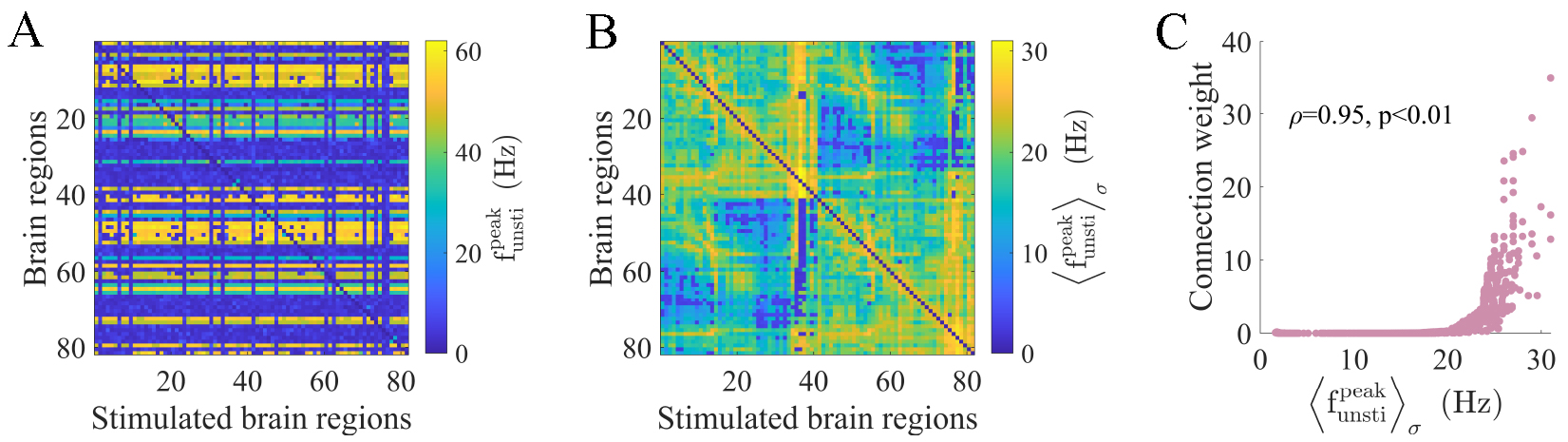

Supplement: S6 Fig — (A) The peak frequency of unstimulated brain regions (funstipeak) (y-axis) under different stimulated brain regions (x-axis) at a large noise amplitude (10−2). The diagonal elements are set to 0. This result corresponds to oscillations before stimulation. (B) The peak frequency of unstimulated brain regions (y-axis) under different stimulation sites (x-axis) averaged across various noise amplitudes that do not induce oscillations before stimulation (〈funstipeak〉σ). (C) The positive Spearman correlation (ρ = 0.95, p < 0.01) between the matrix in (B) and the structural network is similar to Fig 4E. (JPG) [file pcbi.1010866.s006.jpg]

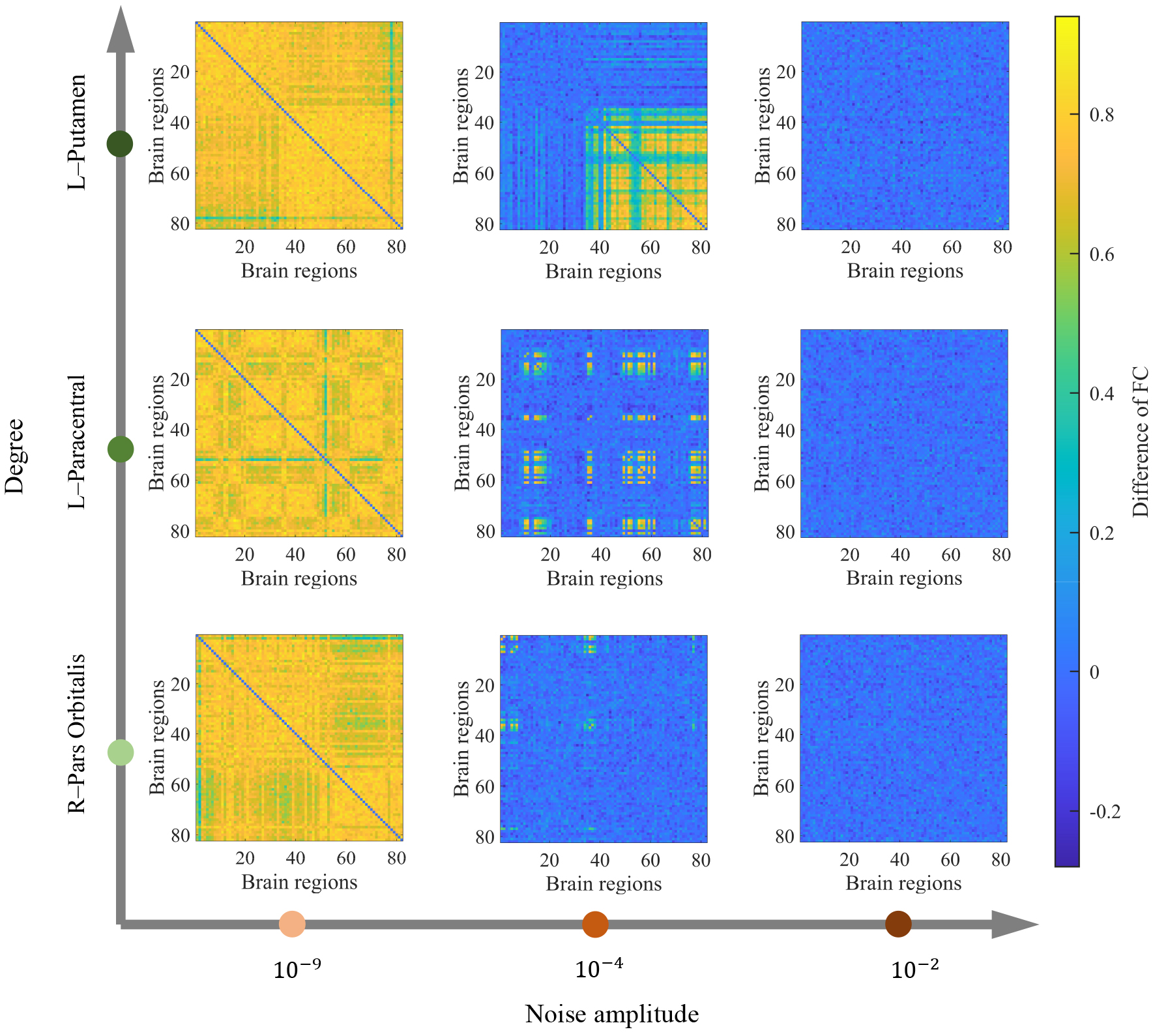

Supplement: S7 Fig — Different Brain regions R-Pars Orbitalis (low degree), L-Paracentral (moderate degree) and L-Putamen (high degree) are stimulated at low (10−9), moderate (10−4) and high (10−2) noise amplitudes. The matrices represent the differences in functional connectivity networks before and during local stimulation for one realization. (JPG) [file pcbi.1010866.s007.jpg]

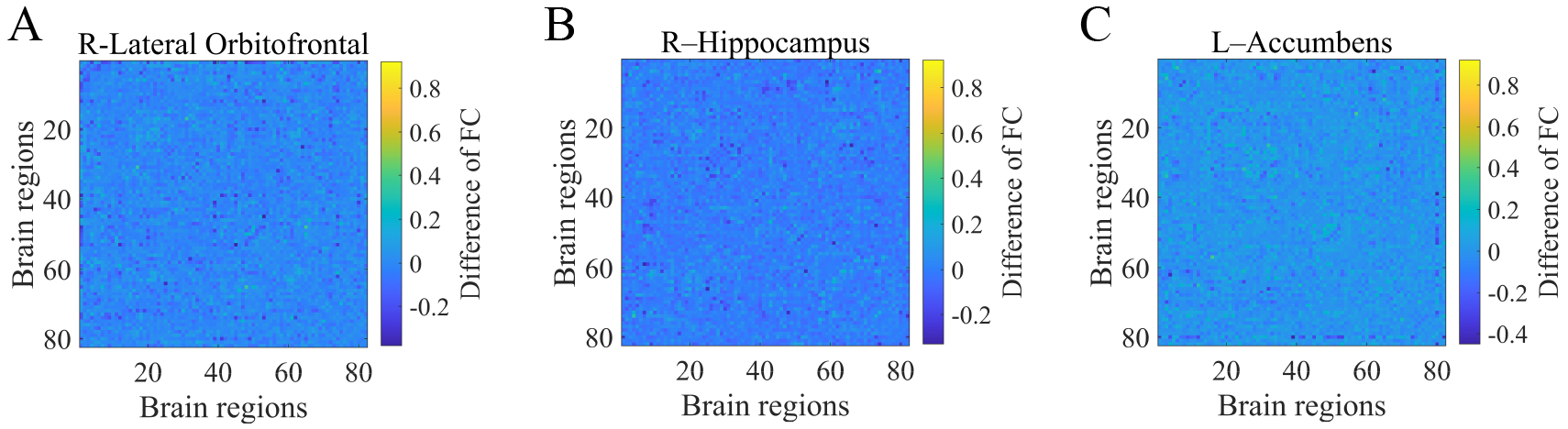

Supplement: S8 Fig — Note that the brain is already in the oscillatory state before stimulation for these examples. Results show small connectivity changes similar to Fig 5. (A) R-Lateral Orbitofrontal. (B) R-Hippocampus. (C) L-Accumbens. (JPG) [file pcbi.1010866.s008.jpg]

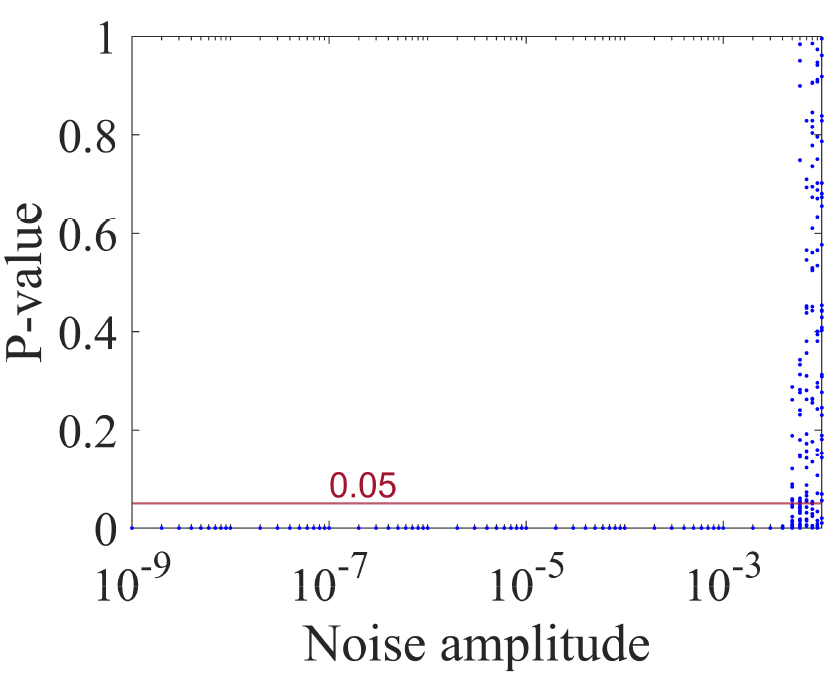

Supplement: S9 Fig — The blue dots at each noise amplitude represent the 30 realizations of p-values. The red horizontal line indicates the position where the p-value is equal to 0.05. FDR correction was performed for p-values across all noise amplitudes and realizations. (JPG) [file pcbi.1010866.s009.jpg]

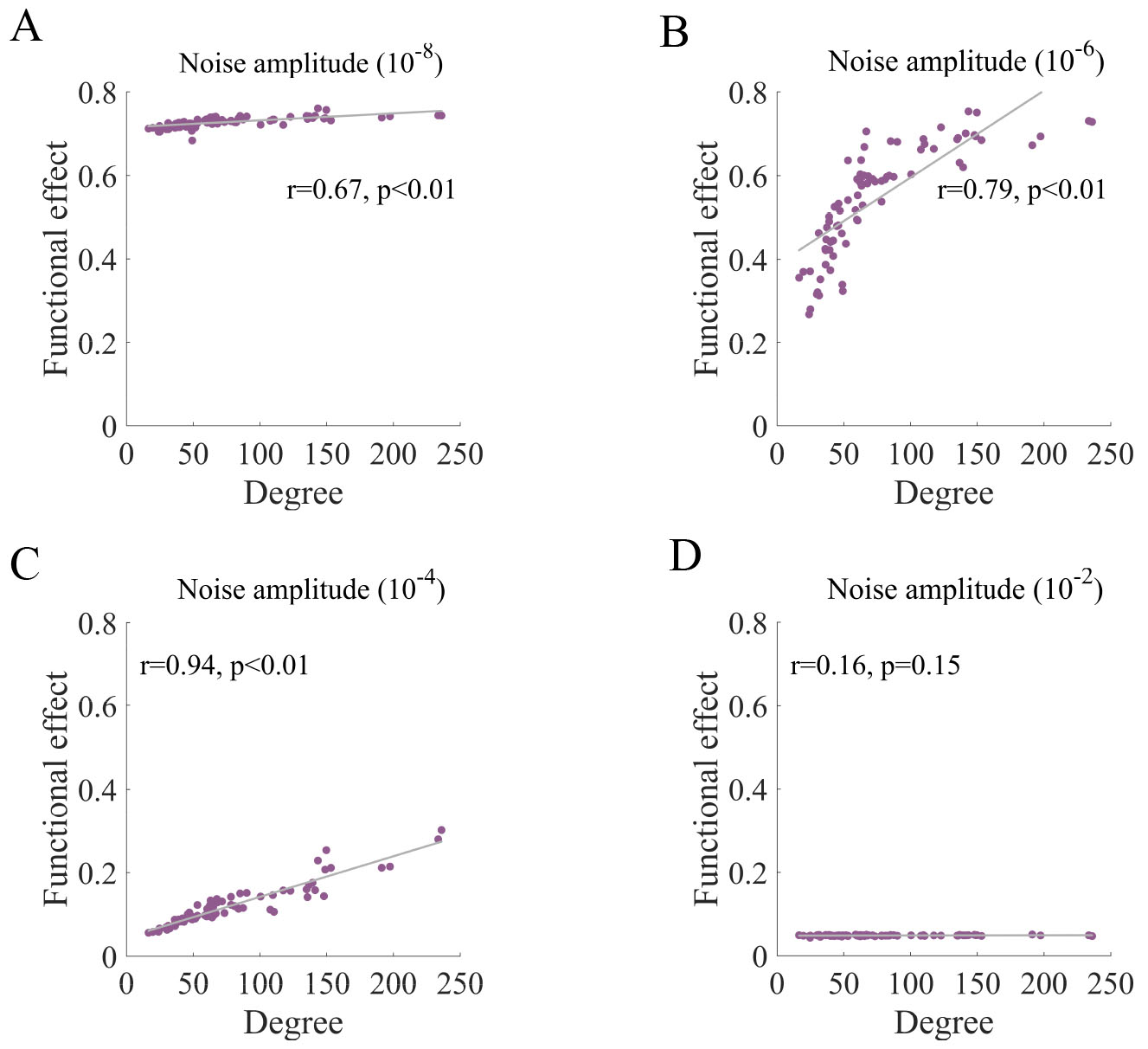

Supplement: S10 Fig — (A) Noise amplitude = 10−8, Pearson’s r = 0.67, FDR-corrected p < 0.01. (B) Noise amplitude = 10−6, Pearson’s r = 0.79, FDR-corrected p < 0.01. (C) Noise amplitude = 10−4, Pearson’s r = 0.94, FDR-corrected p < 0.01. (D) Noise amplitude = 10−2, Pearson’s r = 0.16, FDR-corrected p = 0.15. The gray lines represent the linear fits of data points estimated by ordinary least squares. These results are in line with the trend shown in Fig 6C. (JPG) [file pcbi.1010866.s010.jpg]

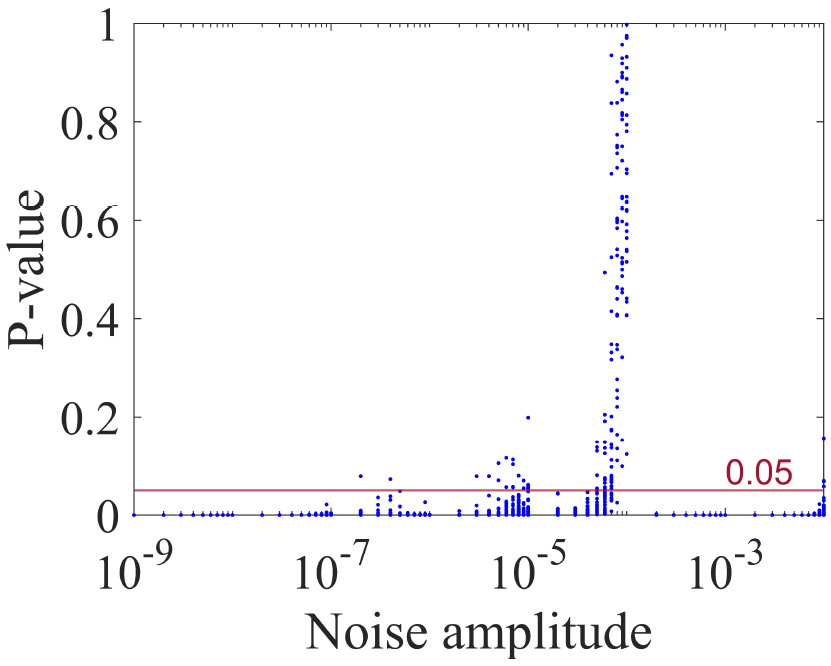

Supplement: S11 Fig — The blue dots at each noise amplitude represent the 30 realizations of p-values. The red horizontal line indicates the position where the p-value is equal to 0.05. FDR correction was performed for p-values across all noise amplitudes and realizations. (JPG) [file pcbi.1010866.s011.jpg]

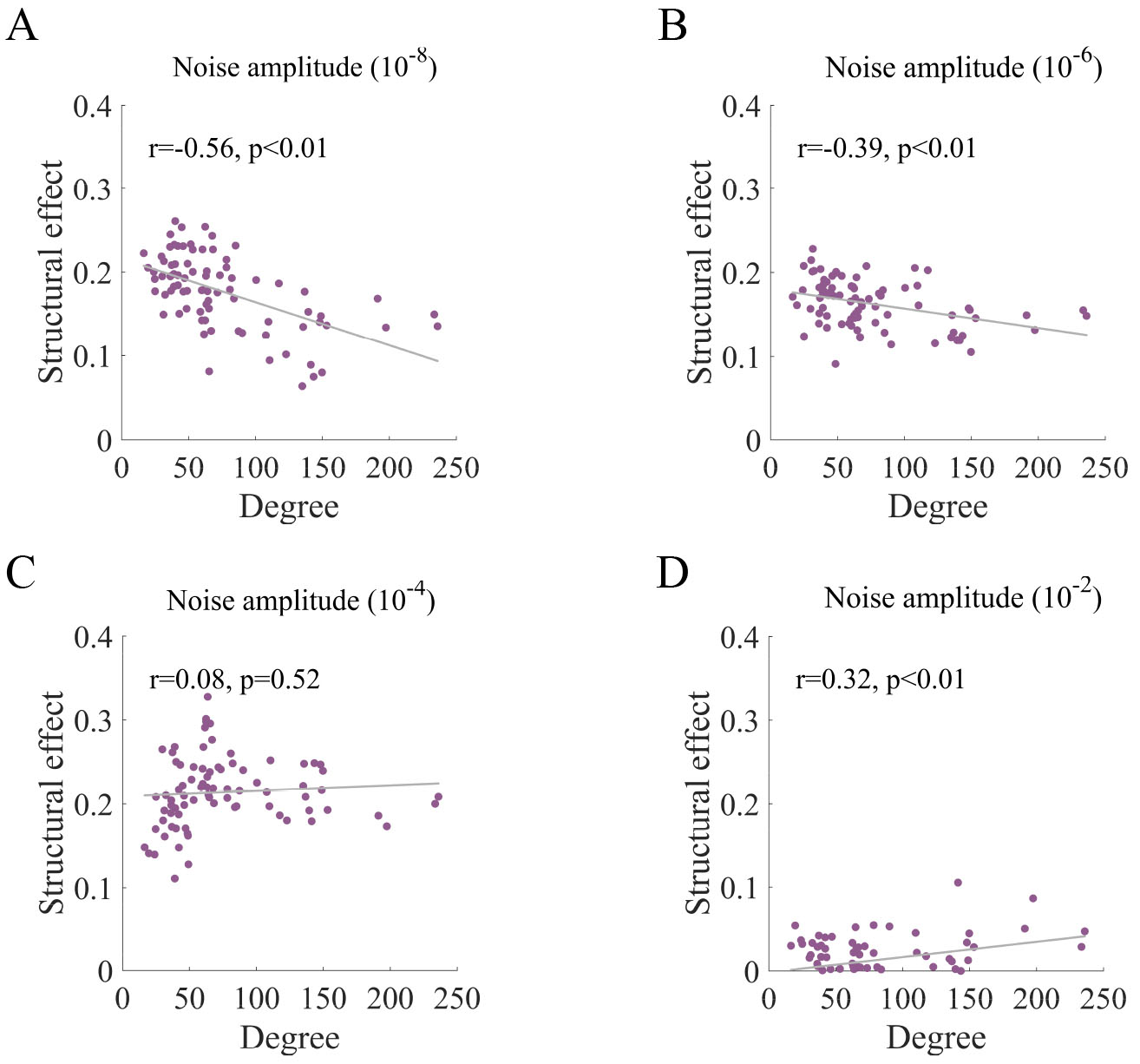

Supplement: S12 Fig — (A) Noise amplitude = 10−8, Pearson’s r = −0.56, FDR-corrected p < 0.01. (B) Noise amplitude = 10−6, Pearson’s r = −0.39, FDR-corrected p < 0.01. (C) Noise amplitude = 10−4, Pearson’s r = 0.08, FDR-corrected p = 0.52. (D) Noise amplitude = 10−2, Pearson’s r = 0.32, FDR-corrected p < 0.01. The gray lines represent the linear fits of data points estimated by ordinary least squares. These results are consistent with the trend shown in Fig 7C. (JPG) [file pcbi.1010866.s012.jpg]

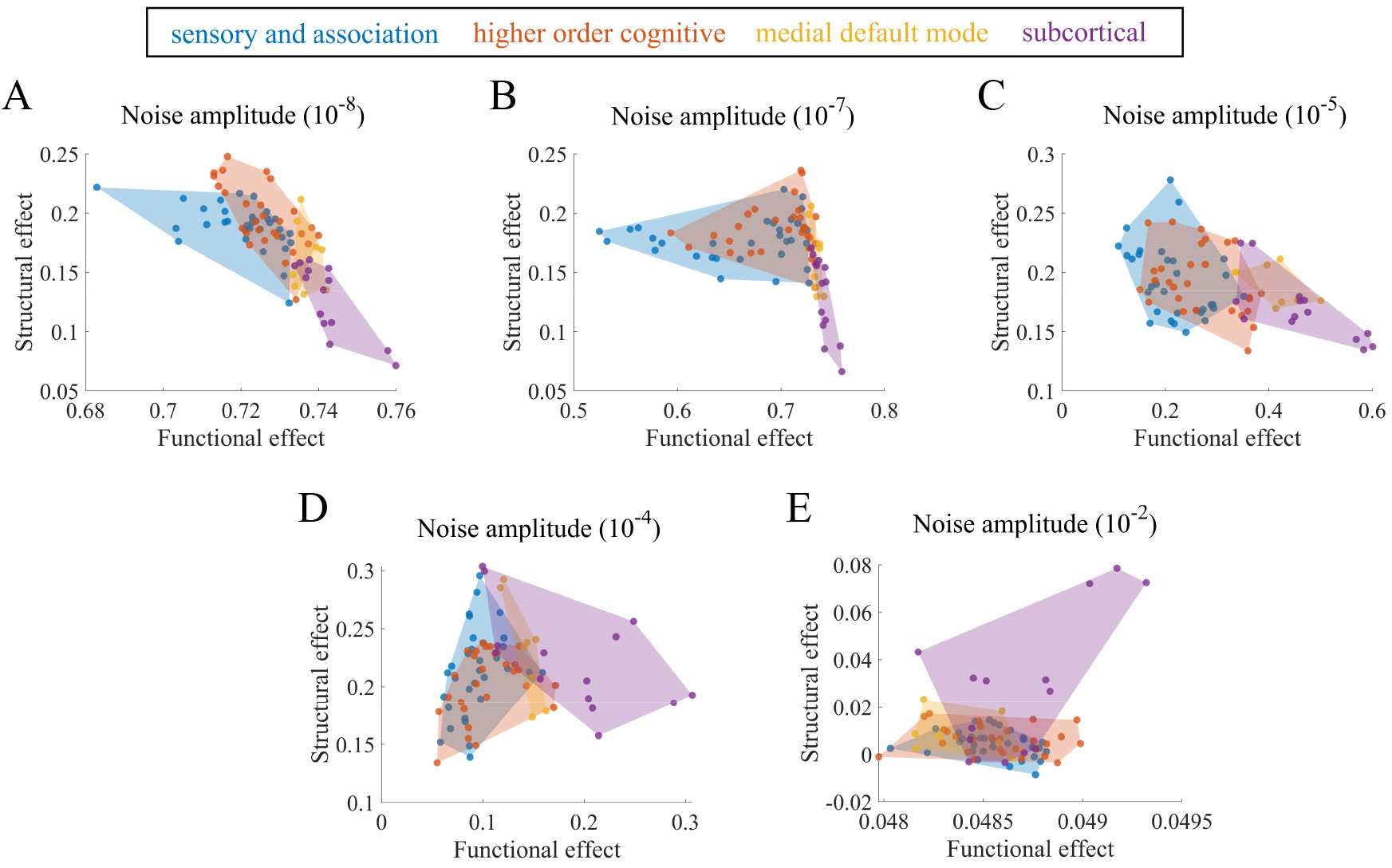

Supplement: S13 Fig — (A) Noise amplitude = 10−8. (B) Noise amplitude = 10−7. (C) Noise amplitude = 10−5. (D) Noise amplitude = 10−4. (E) Noise amplitude = 10−2. Note that stimulated brain regions are grouped into 4 cognitive systems with different colors. The colored areas represent the convex hulls of data points in the systems. The points reflect the measures averaged over 30 realizations. (JPG) [file pcbi.1010866.s013.jpg]
